# Distribution of high- and low-risk human papillomavirus genotypes and their prophylactic vaccination coverage among West African women: systematic review

**DOI:** 10.1186/s43046-023-00196-x

**Published:** 2023-12-07

**Authors:** Rogomenoma Alice Ouedraogo, Ali Kande, Wendyam Marie Christelle Nadembega, Djeneba Ouermi, Théodora Mahoukèdè Zohoncon, Florencia Wendkuuni Djigma, Charlemagne Marie Ragnag-Newende Ouedraogo, Olga Mélanie Lompo, Jacques Simpore

**Affiliations:** 1 Université Joseph KI-ZERBO, Laboratoire de Biologie Moléculaire et de Génétique, P.O. Box 7021, Ouagadougou 03, Burkina Faso; 2 Centre de Recherche Biomoléculaire Pietro Annigoni (CERBA), P.O. Box 364, Ouagadougou 01, Burkina Faso; 3 Université Nazi BONI, P.O Box 1091, Bobo-Dioulasso 01, Burkina Faso; 4 Université Saint Thomas d’Aquin, P.O. Box 10212, Ouagadougou 06, Burkina Faso; 5 Université Joseph KI-ZERBO, UFR SDS, P.O. Box 7021, Ouagadougou 03, Burkina Faso; 6 Centre Hospitalier Universitaire Yalgado Ouedraogo (CHU/YO), P.O. Box 7022, Ouagadougou, Burkina Faso

**Keywords:** HPV, Genotypes, Prevalence, Cervical cancer, HPV vaccine, West Africa

## Abstract

**Introduction:**

The second most deadly gynecological cancer worldwide, cervical cancer is steadily on the rise in sub-Saharan Africa, while vaccination programs are struggling to get off the ground. This systematic review’s aim was to assess the prevalence and distribution of high- and low-risk HPV genotypes in West African women.

**Methods:**

Original studies were retrieved from PubMed/Medline, Embase, Scopus, Google Scholar, and Science Direct. In these studies, Human papillomavirus (HPV) DNA was assessed in cervical samples by polymerase chain reaction (PCR), Hybrid capture, and sequencing. The quality of the articles was assessed and the results were extracted and reviewed.

**Results:**

Thirty-nine studies from 10 West African countries were included for the systematic review including 30 for the pooled analysis. From an overall of 17358 participants, 5126 of whom were infected with at least one HPV genotype, the systematic review showed a prevalence varying from 8.9% to 81.8% in the general population. In contrast, the pooled prevalence of infection was 28.6% (*n* = 3890; 95% CI 27.85–29.38), and HPV-52 (13.3%), HPV-56 (9.3%), and HPV-35 (8.2) were the most frequent. Quadrivalent and nonavalent vaccines covered 18.2% and 55.8% of identified genotypes respectively.

**Conclusion:**

Faced with this growing public health challenge in West Africa, it would be necessary for all its countries to have reliable data on HPV infection and to introduce the nonavalent vaccine. A study of the genotypic distribution of HPV in high-grade precancerous lesions and cervical cancer would be very useful in West Africa.

**Supplementary Information:**

The online version contains supplementary material available at 10.1186/s43046-023-00196-x.

## Introduction

Around the world, numerous epidemiological, clinical, and molecular studies have shown that human papillomavirus (HPV) infection is a necessary risk factor for the development of precancerous lesions and cervical cancer. The second most deadly gynecological cancer among women worldwide [1] and particularly among those aged 15–44 [2], 80% of cervical cancer cases are identified in developing countries [3] and are closely linked to human papillomaviruses [4]. Able to infect the epithelium of the anogenital tract or other mucous membranes [3, 5], HPVs are also thought to be involved in cancers of the vagina, penis, vulva, anus and oropharyngeal cavity [6], and at least 13 high-risk oncogenic HPV (HR-HPV) genotypes [7–19] are highly carcinigenic to humans [6, 20]. Also, some low-grade precancerous lesions induced by certain HPV genotypes can potentially evolve into cervical cancer [21]. Furthermore, some low-risk HPV genotypes (LR-HPV) such as HPV 6 and 11 are responsible for condylomatous lesions (warts) of the anogenital tract and recurrent respiratory papillomatosis (RRP), the most common benign tumor of the larynx [22, 23].

Moreover, cervical cancer appears to be steadily increasing in sub-Saharan Africa [24], and its incidence in West Africa is among the highest in the world [25]. To reduce morbidity and mortality from persistent HPV infection and cervical cancer, HPV-type-specific vaccination is widely recommended [26, 27]. According to the World Health Organization (WHO), at least a third of all HPV-related cancers in Africa could be prevented through full implementation of vaccination [28]. However, the distribution of HPV genotypes can vary from country to country [26, 29] and studies have shown that Africa is home to heterogeneous genotypes [24]. Several meta-analyses have reported that HPV-16, 18, 31, 52, and 58 were the most prevalent in women and that HPV-16/18 were the predominant oncogenic genotypes, responsible for around 70 % of cervical cancer cases worldwide [9, 30–32]. But in sub-Saharan Africa, HR-HPV-16, 18, 35, and 52 were the most common [24]. The licensed prophylactic HPV vaccines, Gardasil® quadrivalent (6/11/16/18), Cervarix® bivalent (16/18) and a nonavalent Gardasil® (6/11/16/18/31/33/45/52/58) have been shown to be safe and effective [10, 33, 34] but their efficacy on cancer prevention could be reduced in populations heavily affected by HR-HPV types other than HPV-16 and 18. The variability of HPV genotype distribution has therefore intensified the debate on HPV vaccine efficacy in Africa particularly in West Africa [11].

In most West African countries, as a result of recurring public health problems such as malnutrition, Human immunodeficiency virus (HIV), and tuberculosis, the vaccination program is struggling to get off the ground [35, 36]. Nevertheless, the vaccination program in some of these countries targets bivalent (16/18) and quadrivalent (6/11/16/18) prophylactic vaccines. However, the nonavalent vaccine would protect against genotypes 6/11/16/18/31/33/45/52/58. Are the genotypes identified in women from the general population, in high-grade precancerous lesions and invasive cervical cancers in West African countries, mostly covered by the vaccines available from these vaccination programs? A systematic review of the prevalence and distribution of HPV genotypes with increased oncogenic risk in West African women therefore seems necessary to better guide thinking on the fight against cervical cancer, whose burden is high. This systematic review’s aim was to assess the prevalence and distribution of high- and low-risk HPV genotypes in West African women.

## Method

### Study design

This study was conducted using The Preferred Reporting Items for Systematic Reviews and Meta-Analyses (PRISMA) [37].

### Search strategy

We conducted a systematic literature review to identify relevant publications reporting the prevalence and distribution of 16 high oncogenic and low-risk HPV genotypes (6/11/16/18/31/33/35/39/45/51/52/56/59/66/68) in West African countries from 2002 to July 31, 2023. Systematic searches in French and/or English were carried out in the PubMed/Medline, Embase, Scopus, Google Scholar, and Science Direct databases. Identified records were downloaded in an appropriate format and linked to Endnote X8 software. Boolean operators "AND" and "OR" were used to link keywords/terms and to retrieve publications from PubMed / Medline databases (NCBI). The keywords used were "HPV and/or HPV and cervical cancer" + "the name of each of the West African countries". In order to limit the search for keywords in the title and/or abstract of articles, a filter was used [PubMed : (tiab)]. Searches with similar terms such as "human papillomavirus", "high-risk human papillomavirus", "HPV infection", "human papillomavirus genotypes", were also conducted. On the basis of crude numerators and denominators available from eligible studies, the crude prevalence of HPV infection was calculated.

### Eligibility criteria and study selection procedure

After consulting the databases, the studies were then selected on the basis of the following criteria: (1) data published in a peer-reviewed scientific journal; (2) complete article with related data available; (3) only patients residing in one of the West African countries; (4) patients from these countries who consulted for gynecological problems or who participated in a screening campaign and were infected with HPV; (5) the results of cervical histology are confirmed for patients with cervical cancer or high-grade precancerous lesions (studies carried out on fixed or fresh biopsies and/or exfoliations); (6) HPV prevalence is calculated with at least five genotypes identified.

In addition, studies eligible for the pooled analysis were required to report on the prevalence and genotypic distribution of oncogenic HPV in women from the countries included, and the molecular diagnosis of HPV based on molecular biology techniques including polymerase chain reaction (PCR), hybrid capture, and sequencing. HPV genotype classification was also taken into account. We systematically excluded journal articles, publishers' correspondence, news items, letters, book chapters, publications in languages other than English/French, and studies whose data were ambiguous or could not be extracted. We also excluded articles dealing with HPV infection in sex workers, homosexuals, HIV-positive populations only, mixed populations with very high HIV positivity, and articles not presenting details of HPV genotype distribution. In addition, for the (comparative) case studies, we considered only HIV-negative populations and cervical swabs taken by health workers.

The search and selection of relevant articles from the databases were carried out by two independent reviewers (TMZ and AK) in order to reduce the risk or minimize the risk of information, selection, and analysis bias. Inclusion of study investigating HPV prevalence and genotype distribution in women by both reviewers was a requirement. In the event of a disagreement over the eligibility of a study, the problem was resolved by discussion and/or consensus with a third reviewer. Thus, the quality of the studies was examined, low-quality studies discarded, and the data from the remaining studies presented using tables for an independent assessment of the methodological quality of each of them. In the event of disagreement between the two evaluators, the intervention of the third evaluator was necessary.

### Data extraction and analysis

Data were extracted from various studies conducted in West African countries for systematic review. For the articles included in our study, the variables extracted were first author, year of data publication, study population, type of clinical sample, type of study or data collection (cross-sectional, prospective, or retrospective), country, inclusion criteria for research study participants, detection methods, number of samples successfully genotyped and genotype results identified. In addition, three eligible mixed studies [12, 25, 38] presented both clear data on general population women and cervical cancer; each of these studies was considered as an independent paper in the analysis. In addition, the overall crude prevalence of HPV infection was determined as the ratio of the total number of women testing positive for at least one HPV to the total number of samples tested, expressed as a percentage. Genotypes were calculated as the ratio of the genotype in question to the total number of genotypes identified, taking into account single and multiple infections. STATA V16 (Statistical software for data science) and Excel 2016.lnk were used for calculations and charting. Confidence intervals (CI) were calculated using Epi info and set at 95%. The Chi-square test was used for comparison and the difference was statistically significant for *P* < 0.05.

### Risk of bias assessment for individual studies

Based on the risk of bias, the methodological quality of the studies was assessed. The risk of bias was assessed using the appropriate Joanna Briggs Institute (JBI) critical appraisal checklists. The JBI critical appraisal tools aim not only to assess the methodological quality of the study but also to determine the extent to which the study has addressed the possibility of bias in its design, conduct, and data analysis [39].

These critical appraisal checklists include specific criteria for identifying scores for different studies. An overall assessment was then performed to decide whether to include or exclude studies [40]. All studies whose score was considered to be of acceptable quality were included in our review. For example, for prevalence studies, the critical appraisal checklist has 9 criteria and therefore the JBI score for these studies is between 0 and 9. Scores 7 to 9 were considered good, scores 4 to 6 as moderate, and scores 1 to 3 as small. The study is considered of acceptable quality if the minimum score was 3.

### Quality assessment of the studies

The quality of included studies in this systematic review was assessed using the JBI critical appraisal for the methodological quality of articles in accordance with the study design. This assessment tool resembles the risk of bias assessment and helps identify studies of acceptable methodological quality. Additional file 1 then presents the PRISMA checklist of the study.

## Results

### Selection of studies for review

**Fig. 1:**
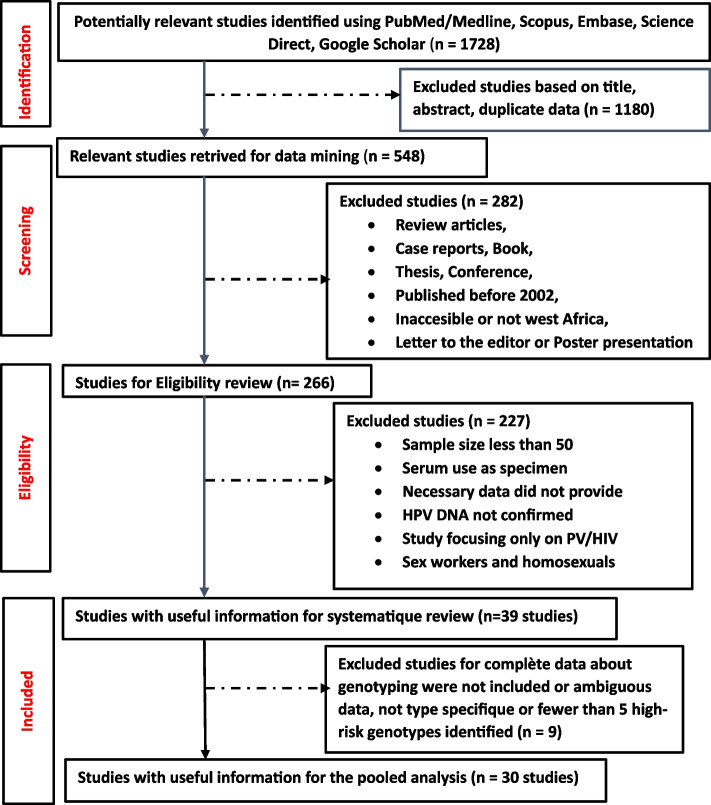
PRISMA flowchart of the search strategy for inclusion in published studies [37]

In this study, a total of 1728 articles were retrieved and for the systematic review, 266 full articles were examined. According to our inclusion criteria, 39 scientific studies focusing on epidemiological studies from 10 West African countries, totaling 17,358 participants from the general female population were included for data extraction. The search results and the number of articles included and excluded are shown in Fig. 1 [37].

### Characteristics of studies included

Data were gathered from studies conducted in West African populations, published between 2002 and 2023, and meeting our selection criteria. Of the 17,358 participants from the general female population, 5126 were infected with at least one of the HPV genotypes (*6/11/16/18/31/33/35/39/45/51/52/56/59/66/68*). Of these 39 scientific studies, 30 studies involving a total of 13596 participants had complete information on the genotypic distribution of HPV and were therefore eligible for the pooled analysis. Twenty-eight studies reported multiple infections with any specific HPV type.

### Prevalence of HPV infection from studies in West African countries and by sample type

This systematic review, based on 39 studies, reports on the prevalence and distribution of HPV genotypes with increased oncogenic risk in West African women. Our analysis of published studies shows that the prevalence of HPV infection is high in this part of Africa. This prevalence and genotypic distribution varies from country to country and according to the patient's state of health. Indeed, among women in the general population who consulted a doctor for gynecological problems, HPV prevalence ranged from 8.9 to 81.8% [41, 42] (Table 1).

**Table 1:** Summary table of original articles on HPV infection in West Africa based on search strategy

	Contributive studies, *N* = 39 studies			Type of study	Average age or age range (years)	Tested, *N*	HPV+		
	References (1st author)	Year	Country				*N*	%	95%CI
General population women	Domfeh et al*.* [43]	2008	Ghana	Cross-sectional	19–57	75	8	10.7	5.08–19.25
	Schulze et al. [44]	2016	Ghana	Cross-sectional	–	165	55	33.3	26.30–41.10
	Obiri et al. [45]	2017	Ghana	Comparative cross-sectional	44.3 ± 12.8	169	72	42.5	35.30–50.15
	Krings et al. [13]	2019	Ghana	Cross-sectional observational	˃ 15	500	104	20.8	17.41–24.53
	Debrah et al. [26]	2021	Ghana	Cross-sectional	21–76	317	138	43.5	37.90–48.60
	Donkoh et al. [27]	2022	Ghana	Cross-sectional observational	18–93	500	186	37.2	32.90–41.40
	Thomas et al. [46]	2004	Nigeria	Cross-sectional	≥ 15	932	245	26.3	23.54–29.19
	Akarolo-Antony et al. [47]	2014	Nigeria	Cross-sectional	31–42	275	101	37	31.18–42.55
	Fadahunsi et al. [48]	2013	Nigeria	Cross-sectional	18–68	111	24	21.6	14.71–29.99
	Manga et al. [49]	2015	Nigeria	Cross-sectional observational	39.6 ± 10.4	208	100	48.1	41.34–54.87
	Adebamowo et al. [50]	2017	Nigeria	Cross-sectional	≥ 18	535	114	21.3	17.99–24.93
	Modibo et al. [41]	2017	Nigeria	Cross-sectional	30–65	113	10	8.9	4.58–15.20
	Nejo et al. [14]	2018	Nigeria	Cross-sectional	23–77	295	55	18.6	14.51–23.39
	Ogah et al. [15]	2019	Nigeria	Cross-sectional	15–60	200	23	11.5	7.61–16.49
	Nejo et al. [51]	2019	Nigeria	Cross-sectional	23–77	295	48	16.3	12.38–20.81
	Ashaka et al. [42]	2022	Nigeria	Cross-sectional	18–65	165	135	81.8	75.38–87.15
	Cosmas et al. [52]	2022	Nigeria	Cross-sectional	9–20	205	27	13.2	9.04–18.33
	Okoeguale et al. [53]	2022	Nigeria	Cross-sectional	37–13	145	24	16.6	11.15–23.27
	Piras et al. [54]	2011	Benin	Cross-sectional	15–70	427	142	33.2	28.91–37.83
	Gandekon et al. [55]	2020	Benin	Cross-sectional	19–60	247	81	32.8	27.15–39.30
	Capo-Chichi et al. [16]	2016	Benin	Cross-sectional case-control	20–60	86	20	23	15.24–33.04
	Chabi et al. [56]	2019	Benin	Retrospective comparative analysis	18–59	234	80	34	28.32–40.44
	Ouedraogo et al. [17]	2011	Burkina Faso	Cross-sectional	15–63	300	73	24.3	19.73–29.43
	Ouattara et al. [18]	2019	Burkina Faso	Cross-sectional	30.7 ± 7.3	234	48	20.6	15.70–26.04
	Djigma et al. [57]	2020	Burkina Faso	Cross-sectional	15–63	238	117	49.2	42.84–55.50
	Ouedraogo et al. [58]	2020	Burkina Faso	Cross-sectional	15–76	1321	468	35.4	32.88–38.04
	Kabré et al. [59]	2022	Burkina Faso	Cross-sectional	18–40	100	23	23	15.54–31.99
	Wall et al. [7]	2005	Gambia	Cross-sectional	15–54	710	95	13.4	10.96–16.11
	Bah Camara et al. [60]	2018	Gambia	Cross-sectional	20–49	232	28	12	8.33–16.74
	Horo et al. [61]	2022	Ivoiry Coast	Cross-sectional	43.33	250	85	34	28.33–40.04
	XI et al. [62]	2002	Senegal	Cross-sectional	≥ 35	2065	366	18	16.12–19.41
	Mbaye et al. [63]	2014	Senegal	Cross-sectional	≥ 18	936	214	22.8	20.26–25.64
	Faye et al. [64]	2022	Senegal	Prospective, descriptive, and comparative	–	198	128	64.6	57.79–71.07
	Dolou et al. [65]	2021	Togo	Cross-sectional	17–61	238	85	35.7	29.82–41.95
	Kuassi-Kpede et al. [66]	2021	Togo	Cross-sectional	17–67	240	128	53.3	47.00–59.58
Mixed studies (general women's population)	Keita et al. [25]	2009	Guinea Conakry	Cross-sectional	18–64	831	422	50.8	47.38–54.17
	Nartey et al. [12]	2023	Ghana	Cross-sectional	≥ 18	201	92	45.8	38.97–52.69
	Okolo et al. [38]	2010	Nigeria	Retrospective	≥ 15	932	245	26.3	23.54–29.20
	Zohoncon et al. [19]	2016	Burkina Faso, Togo, Benin, Niger, Ivory Coast	Cross-sectional Descriptive	15–65	2133	717	33.6	31.63–35.64
	Overall		10 Countries	–	–	17358	5126		–

### Overall prevalence of HPV infection in West Africa

Of the 30 studies eligible for the pooled analysis, totaling 13596 participants, the pooled analysis indicated an overall prevalence of HPV infection of 28.6% (3890/13596 ; [95%, CI: 27.85–29.38]).

**Fig. 2:**
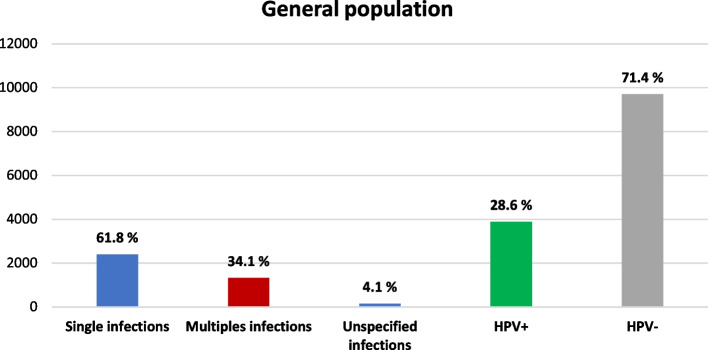
Frequency of single and multiple infections and prevalence of HPV infection in West African women

In addition, among the HPV-infected women identified and classified according to risk category by the original studies, single and multiple infections were 61.8% (2402/3890) and 34.1% (1328/3890) respectively, making a total of 95.9% (3730/3890) classified single and multiple HPV infections versus 4.1% (160/3890) unspecified HPV infections (Fig. 2).

### Distribution of HR-HPV genotypes among women in West Africa

Genotypes *HPV-6, 11, 16, 18, 31, 33, 35, 39, 45, 51, 52, 56, 59, 66, 68* were sought in our systematic review. From the 10 West African countries included in our study and according to our inclusion criteria, the systematic review of the 30 studies eligible for the pooled analysis on women from the general population showed that the distribution of HPV genotypes had a geographical variation (Table 2). Diagnosis using molecular biology techniques, notably real-time multiplex PCR, nested multiplex PCR, hybrid capture, and sequencing, has shown that HPV 16 and 18, which are generally frequent in cases of cervical cancer, are giving way to other genotypes in women from the general population in most West African countries (Table 2).

**Table 2:** Distribution of high-risk oncogenic HPV (HR-HPV) genotypes from original articles retrieved for the systematic review

Study population	References (1st author)	Years	Country	Eight most frequent HR-HPV genotypes in descending order
General population women	Schulze et al. [44]	2016	Ghana	52/45/18/51/58/59/35/66
	Obiri et al. [45]	2017	Ghana	35/33/58/56/52/18/39/68
	Krings et al. [13]	2019	Ghana	18/58/52/68/66/51/35/56
	Debrah et al. [26]	2021	Ghana	52/58/68/66/35/16/45/18
	Donkoh et al. [27]	2022	Ghana	52/56/35/18/66/68/58/51
	Thomas et al. [46]	2004	Nigeria	35/16/31/58/56/66/45/18
	Adebamowo et al. [50]	2017	Nigeria	52/68/18/35/56/33/39/45
	Modibo et al. [41]	2017	Nigeria	18/51/52/16/35/56/58/66
	Nejo et al. [51]	2019	Nigeria	31/35/16/18/33/66/58/52
	Cosmas et al. [52]	2022	Nigeria	52/51/18/58/66/31/45/68
	Okoeguale et al. [53]	2022	Nigeria	16/45/18/35/52/58
	Piras et al. [54]	2011	Benin	59/35/16/18/58/45/56/33
	Capo-Chichi et al. [16]	2016	Benin	66/33/56/58/18/59/16/45
	Chabi et al. [56]	2019	Benin	52/58/51/35/45/66/68/39
	Gandekon et al. [55]	2020	Benin	45/35/52/51/58/39/68/59
	Ouattara et al. [18]	2019	Burkina Faso	52/66/68/39/51/18/31/35
	Djigma et al. [57]	2020	Burkina Faso	18/52/58/35/16/31/45/51
	Ouedraogo et al. [58]	2020	Burkina Faso	56/52/66/59/39/51/18/35
	Kabré et al. [59]	2022	Burkina Faso	52/35/16/31/33/45/51/56
	Horo et al. [61]	2022	Ivoiry Coast	68/52/56/35/45/58/51/31
	XI et al. [62]	2002	Senegal	16/58/18/33/52/31/51/59
	Mbaye et al. [63]	2014	Senegal	31/52/68/66/45/16/51/56
	Faye et al. [64]	2022	Senegal	56/66/68/52/31/16/58/18
	Dolou et al. [65]	2021	Togo	31/52/68/66/58/56/51/45
	Kuassi-Kpede et al. [66]	2021	Togo	56/51/31/52/35/18/66/58
	Wall et al. [7]	2005	Gambie	16/35/58/33/18/31/52/45
	Bah Camara et al. [60]	2018	Gambie	52/51/58/66/16/35/56
Mixed studies (general women’s population)	Keita et al. [25]	2009	Guinea	16/45/52/66/33/58/35/18
	Nartey et al. [12]	2023	Ghana	66/52/35/56/68/51/39/45
	Okolo et al. [38]	2010	Nigeria	16/35/31/56/58/45/18/51

**Fig. 3:**
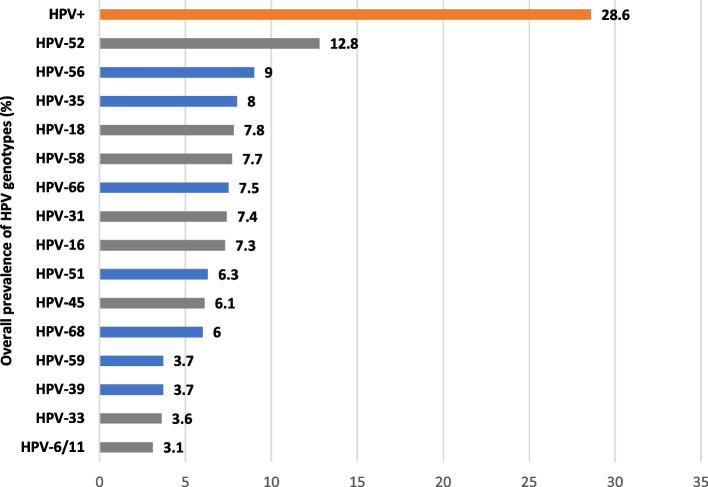
Distribution of HPV genotypes among study women in West Africa

On the other hand, of the 3890 HPV-infected participants included in the pooled analysis, the collective analysis of data from the 30 eligible studies showed that the most frequent HR-HPV genotypes were *HPV-52, 56, 35, 58, 18, 66, 31, 16, 51, 45, 68, 59, 39, 33* respectively for women in the general population, with a prevalence ranging from 13.3 to 3.7% (Table 3). This genotypic distribution therefore varied with the nature of the clinical sample, i.e., the stage of HPV infection. In addition, low-risk oncogenic genotypes (LR-HPV) such as *HPV-6/11* were the least frequent among the 3890 HPV-infected women from the general population (Fig. 3).

**Table 3:** Overall prevalence of 14 HR-HPV in women from the general population based on collective analysis

HPV	Number	Percentage
HPV-16	340	7.5
HPV-18	363	8
HPV-31	347	7.7
HPV-33	170	3.7
HPV-35	374	8.2
HPV-39	174	3.8
HPV-45	285	6.3
HPV-51	293	6.5
HPV-52	601	13.3
HPV-56	423	9.3
HPV-58	361	8
HPV-59	174	3.8
HPV-66	351	7.7
HPV-68	279	6.2
Genotypes cumulation	4535	100
HPV+	3890	28.6
Total staff	13596

### Coverage rates of HPV genotypes by licensed HPV vaccines based on the pooled analysis

**Fig. 4:**
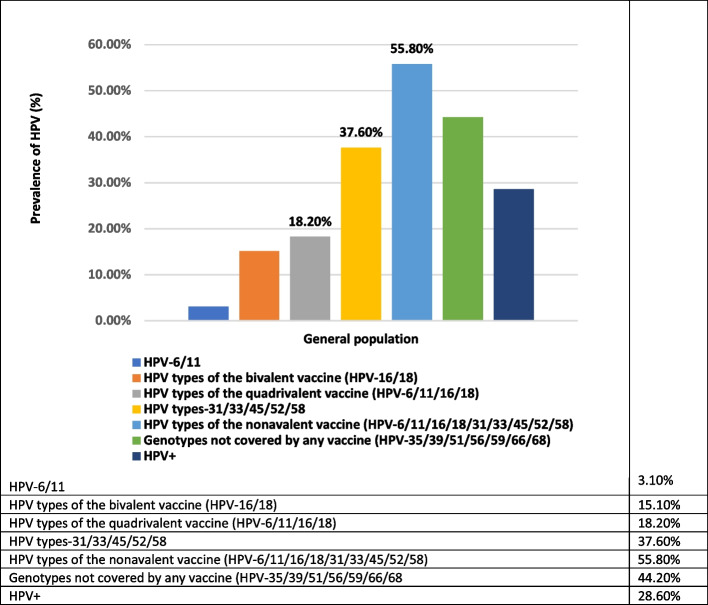
HPV vaccine genotype coverage rates based on the pooled analysis

The bivalent (*16/18*), quadrivalent (*6, 11, 16, 18*), and nonavalent (*6, 11, 16, 18, 31, 33, 45, 52, and 58*) HPV prophylaxis vaccines available offer an alternative for the prevention of HPV infection, the morbidity of which is high. But some genotypes with oncogenic risks are not covered by these licensed vaccines and are unfortunately found in our populations. Thus, of all the genotypes identified in the 3890 HPV-infected women in this study, HPV-6/11 was represented in 3.1% of women in the general population, while the bivalent vaccine (*HPV-16/18*) covered 15.1 % of the genotypes identified. In addition, the other high-risk oncogenic HPVs (*HPV-31/33/45/52/58*) included in the nonavalent vaccine had a prevalence of 37.6 %. Thus, the nonavalent vaccine had a coverage rate of 55.8%. For genotypes not covered by an HPV vaccine (*HPV-35/39/51/56/59/66/68*), their prevalence was 44.2% (Fig. 4).

## Discussion

In the mechanism of cervical cancer control, there is a need for up-to-date data on the prevalence and distribution of HPVs, particularly HR-HPVs, to assess the potential impact of licensed vaccines on cervical cancer prevention, and to identify effective strategies for cancer control programs.

Based on 39 studies, this systematic review reports the prevalence and distribution of HPV genotypes among women in West Africa.

Thus, the systematic review reported a high prevalence of HPV infection in West Africa and both geographic and specific variability in the type of clinical sample or health status of women. It ranged from 8.9 to 81.8% in women from the general population and was similar to previous studies from Sub-Saharan Africa, which reported a variation of 10.7 to 97.2% for HPV infection [24]. Previous studies have reported a geographical variation in HPV infection around the world [9, 21], with high prevalences explained by the weakening of the infected person's immune system. Indeed, certain immunodepressions such as HIV infection constitute a major public health problem. Yet 70% of people living with HIV lived in Africa in 2012, and in sub-Saharan Africa, women and girls (of all ages) account for 63% of all new HIV infections [67]. Weakening of the immune system would therefore constitute an increased risk factor for HR-HPV infection and persistence [8, 16, 45, 50, 64, 68–71]. This geographical variation in the prevalence of HPV infection in West Africa, as in other parts of the world, suggests the need to develop strategies for the prevention and treatment of HPV infection based primarily on women's health status. As for the overall prevalence of HPV infection, collective analysis of data extracted from 10 West African countries indicated an overall prevalence of 28.6%. This result was close to that of the cross-sectional study by Zohoncon et al. in 2020, which reported a prevalence of 33.6% for oncogenic HR-HPV infection among women in the general population from 5 West African countries (Benin, Burkina Faso, Côte d'Ivoire, Niger, Togo) [19]. It is also close to the overall prevalence of 34% found by Seyoum et al., in Sub-Saharan Africa [24]. On the other hand, this overall prevalence was lower than the 50.5% reported in 2015 by Ogembo et al., through a robust meta-analysis in Africa [9]. This wide variation in the prevalence of HPV infection could be explained by the difference in sample sizes with diagnosed precancerous lesions included in each study, complicating the true estimate of pooled prevalence.

The HR-HPV genotypes targeted by our analysis were *HPV-16, 18, 31, 33, 35, 39, 45, 51, 52, 56, 59, 66, 68.* This systematic review indeed found that the prevalence and distribution of HR-HPV in West Africa varied with several factors. In fact, collective (pooled) analysis of data from the 13596 women involved indicated that among the 3890 women infected with HPV, the most frequent genotypes were *HPV-52, 56, 35, 58, 18, 66, 31, 16, 51, 45, 68, 59, 39, 33* respectively for women in the general population. This distribution seems to confirm the observation made by Seyoum et al., in 2022, who reported that the distribution of HR-HPV genotypes in West African countries was different from that in Southern and East African countries. Genetic factors could therefore explain this varied distribution of HPV genotypes in women and indicate the need for region-specific control programs.

In addition, HPV-52 frequently appeared among the top five genotypes in most of the West African countries included in our study. Its high frequency in the general female population would indicate with previous studies that HPV 52 is very common in West Africa [25, 26, 50, 55, 58, 60, 61, 63, 65].

Moreover, in several studies conducted in West Africa, it was frequently identified in women with cervical cancer [1, 4, 12, 25, 72–74]. However, it is not included in the bivalent/quadrivalent vaccines introduced in the vaccination program of some West African countries.

Available licensed HPV vaccines should prevent cervical and other genital cancers. Given the impossibility of measuring their direct efficacy against cancer, for both ethical and time-related reasons, prevention of high-grade cervical intraepithelial lesions (CIN2,3) would be the best substitute criterion for protection. The quadrivalent Gardasil® vaccine (Merck; Whitehouse Station, NJ, USA), approved by the US Food and Drug Administration (FDA) in 2006, offers primary protection against the most common oncogenic genotypes (types 6/11/16/18), while the bivalent Cervarix vaccine approved at 2009 targets genotypes 16/18. These two vaccines are mainly used in West Africa. However, the nonavalent Gardasil®9 vaccines approved in 2014 offer protection against oncogenic *HPV 6/11/16/18/31/33/45/52/58*. In May 2013, 45 mostly developed countries had introduced HPV vaccination worldwide [75, 76].

Interesting data on the efficacy of HPV vaccination is emerging from numerous studies comparing vaccinated and unvaccinated subjects regarding persistent HPV infection, genital warts, and precancerous lesions [77–81].

In West Africa, thanks to the support of GAVI (Global Alliance for Vaccines and Immunization), several countries such as Senegal, Niger, Ghana, Sierra Leone, Gambia, Mali, Liberia, Côte d'Ivoire, Benin, Burkina Faso, and Togo, have committed to this dynamic through HPV vaccine demonstration programs or national programs [78, 81, 82]. This commitment would require a particular focus on the choice of prophylactic vaccines that cover the most frequent genotypes for the vaccination program. Indeed, in this pooled analysis, among all HPV-infected women, the eight most frequent genotypes were *HPV-52, 56, 35, 58, 18, 66, 31, 16*, totaling 5 HPV types included in Gardasil®9.

In addition, the *HPV-6/11* types included in the vaccines were poorly represented in these women. However, in this pooled analysis, among women from the general population, vaccination coverage was only 15.1% of HPV types with the bivalent Cervarix vaccine (*HPV-16/18*) and 18.2% of genotypes identified with the quadrivalent Gardasil® vaccine. In addition, 37.6% of HPV genotypes (*HPV-31/33/45/52/58*) included in the nonavalent were other than *HPV-6/11/16/18*. According to WHO estimates, the prevalence of *HPV-16/18* in West Africa is 4.3% for women with normal cytology and 55.6% for those with cancer [2]. This difference could be explained by the lack of clear specification of the types of precancerous lesions identified by Visual Inspection with Acetic Acid and Lugol’s (VIA/VILI) in some studies of women from the general population.

Of the three licensed vaccines, the nonavalent Gardasil®9 clearly showed the widest coverage of the genotypes identified in women from the general population, with a prevalence of 55.8%.

This result is in line with previous studies that also found wider coverage of HPV genotypes by the nonavalent vaccine in West Africa [26, 38, 45, 59, 65, 83, 84]. According to some authors, these vaccines may have some cross-protection against other less common HR-HPV types [85, 86]. The introduction of the nonavalent would therefore gain an additional 37.6% coverage among women in the general population. In addition, an observational study conducted in Ghana by Krings et al., reported clearance of certain HPV genotypes after four years, notably low-risk HPV, but the persistence of *HPV-16, 18, 35, 39, 51, 52, 58, 68*, with HPV 68 in CIN 2 and HPV 16 in invasive cervical cancer [13].

In view of these results and the predominance of high-risk oncogenic HPV genotypes identified in the general female population, but especially in cases of high-grade precancerous lesions (CIN 2,3) and cancer, the introduction of the nonavalent vaccine in West African countries would be an excellent way of preventing cervical cancer, the silent killer of our populations.

### Study limits

The major contribution of this systematic review is the evaluation of 13596 women from 10 West African countries, 26.8% of whom were infected with HPV, but the applicability of these data is limited. Indeed, this study has limitations such as the small number of countries included (only 10 countries out of a total of 16); the inclusion of cross-sectional studies and their inherent risk of bias; the highly variable sample size for the studies included, which may interfere with the nature of the study’s representativeness; the lack of representativeness of HR-HPV prevalence due to the variation in the different genotyping methods used; the absence of age-specific data (the majority of studies reported mean or median age) to estimate the appropriate age for HPV molecular screening and cervical cancer screening. These various factors should be taken into account as far as possible in the design of future studies on the African continent and in West Africa in particular.

## Conclusion

With a high prevalence in West Africa, HPV infection is a public health problem. This prevalence varies not only from one country to another but also within the same country. The same applies to the distribution of high-risk oncogenic genotypes, involved in the development of cervical cancer. In order to implement a more relevant vaccination program, all West African countries would need reliable data on HPV infection, including an expression of prevalence according to the health status of the infected person. Establishing the genotypic distribution of HR-HPV in cases of high-grade precancerous lesions and histologically confirmed cervical cancer in West African countries would be judicious to better guide decisions relating to the fight against this silent killer through vaccination. Nevertheless, the majority of these countries could introduce HPV vaccination into their national public health strategy as part of a comprehensive approach to cervical cancer prevention and control. Vaccination programs will also benefit from the introduction of the nonavalent vaccine (Gardasil 9), which includes the majority of HR-HPV genotypes frequently identified in the African population. A revision of the cost of the vaccine for resource-limited countries would enable full implementation of vaccination worldwide. The inclusion of genotypes not covered by existing vaccines by pharmaceutical firms would also be of great public health interest.

## Supplementary Information

**Additional file 1.**

## Data Availability

The datasets generated during and/or analyzed during the current study are available from the corresponding author on reasonable request.

## Abbreviations

HPV: Human papillomavirus
HR-HPV: High-risk HPV
LR-HPV: Low-risk HPV
RRP: Recurrent respiratory papillomatosis
PCR: Polymerase chain reaction
WHO: World Health Organization
HIV: Human immunodeficiency virus
CIN: Cervical intraepithelial lesions
GAVI: Global Alliance for Vaccines and Immunization
VIA/VILI: Visual Inspection with Acetic Acid and Lugol’s
CI: Confidence interval
PRISMA: Preferred Reporting Items for Systematic Reviews and Meta-Analyses
JBI: Joanna Briggs Institute
